# Differences in playing experience, anthropometry and performance measures between Under 16 schoolboy rugby players classified as starters or non-starters: A comparative cross-sectional study

**DOI:** 10.17159/2078-516X/2025/v37i1a19947

**Published:** 2025-06-15

**Authors:** M Chiwaridzo, C von der Heiden, FE Kamba, NS Mkumbuzi

**Affiliations:** 1 Department of Occupational and Physiotherapy, Faculty of Health Sciences and Veterinary Medicine, University of Namibia, Windhoek, Namibia; 2 Rehabilitation Unit, Department of Primary Health Care Sciences, Faculty of Medicine and Health Sciences, University of Zimbabwe, Harare, Zimbabwe; 3 Sport, Exercise and Rehabilitation Department, Northumbria University, Newcastle, United Kingdom; 4 Department of Human Movement Science, Nelson Mandela University, Qheberha, South Africa

**Keywords:** rugby, anthropometrics, performance characteristics, Zimbabwe, playing experience

## Abstract

**Background:**

Schoolboy rugby is becoming increasingly popular around the world. Although sports participation is commendable because of its health benefits, the involvement of schoolchildren in highly competitive rugby continues to stimulate research interest. Questions regarding schoolboys possessing the requisite qualities/skills are inevitable and should be addressed scientifically.

**Objectives:**

The purpose of this study was to determine potential differences in playing experience, anthropometry, and performance measures (physiological characteristics and game-specific skills) between starting and non-starting players in schoolboy rugby.

**Methods:**

A cross-sectional design was utilised with 71 rugby players playing Under 16 rugby. The participants came from three secondary schools based in Harare, Zimbabwe, known for playing highly competitive rugby. The participants were measured for playing experience, anthropometry and performance measures such as speed, agility, upper-and-lower-muscular strength/power, muscle flexibility, prolonged high-intensity intermittent running ability, tackling, passing, and catching abilities.

**Results:**

The starters had more playing experience in competitive schoolboy rugby than non-starters (p<0.001). The Wall Sit Leg Strength test for lower-limb isometric strength (p=0.01), Yo-Yo Intermittent Recovery Level 1 test for prolonged high-intensity intermittent running ability (p=0.03), Tackling Proficiency test (p=0.02*)*, Passing Ability Skills test (p=0.01), and Passing-for-Accuracy over 7 m test (p<0.001) discriminated starters from non-starters. The starters showed superior scores compared to the non-starters.

**Conclusion:**

These results indicate that the qualities/skills are better developed among starters and that further training by coaches is needed among the non-starters.

Rugby union (“rugby”) is a well-recognised contact team sport played worldwide, including in Africa. Recently, due to the growing talent identification initiatives, rugby has become increasingly popular among young people in schools and academies.^[1,2]^ Consequently, junior rugby has become increasingly organised over the years, with highly competitive matches commencing at the Under 16 (U16) age category.^[3]^ Although sports participation is commendable, young people’s involvement in highly competitive rugby still in school has stirred considerable research interest and sometimes controversy. Questions regarding schoolboys’ possessing requisite qualities/skills against the perceived high risk associated with the sport are surfacing.^[4]^ This acknowledges the physical nature of the sport, requiring participants to possess adequate playing experience, suitable physical and physiological attributes, as well as rugby-specific skills necessary for safe and effective competition.^[1]^ Therefore, studies investigating the qualities/skills of schoolboy players involved in competitive rugby are warranted. Such studies are limited, particularly in low- and middle-income countries like Zimbabwe, despite the presence of an established and reputable schoolboys’ rugby programme league.

Regardless of age category, rugby is characterised by repeated performance of physically demanding tasks such as high-intensity sprinting, tackles, scrummaging, rucks and mauls.^[5]^ Literature suggests that age-grade rugby players should exhibit appropriate anthropometric features and adequate speed, endurance, agility, and muscular strength/power essential for peak performance and match success.^[1]^ Additionally, a recent review highlighted that game skills such as passing, catching ability, and tackling represent the core skills of the sport and should be considered when identifying talent.^[6]^ As such, several studies have linked superior scores for the aforementioned physiological characteristics and rugby-specific game skills with elite performances or higher playing standards.^[7,8]^ These findings are from studies comparing participants playing rugby at different levels of competitive standards (*elite vs. sub-elite*).^[7,8]^ However, there is limited understanding of the qualities/skills differentiating elite schoolboy rugby players by playing status (*starters vs. non-starters*). Such studies may facilitate understanding the qualities/skills that coaches consider for team selection and the need for training development for the elite players. Team selection represents one of the most important decisions coaches make regularly. Therefore, players considered regular starters should represent a cohort of players with the qualities/skills coaches perceive essential for team success.

Specifically, there is limited research investigating cross-sectional differences in playing experience, anthropometric features, and performance measures (physiological characteristics and rugby-specific game skills) of elite schoolboy rugby players by playing status. The few studies presented equivocal findings largely because of differences in sports codes, sample population, test batteries utilised, and operational definitions of “starters”.^[9–12]^ Nevertheless, the general notion in literature is that regular starters outperform non-starters in many of the aforementioned variables. For example, among 53 Under 14 (U14) (13.2±0.6 years) rugby league players, Gabbett^[7]^ found that starters had superior playing experience and aerobic endurance than non-starters. In that study, starters were players selected to play in the first game of the junior rugby league competition. However, for the U16 players, the same study showed that only linear speed tests (10m, 20m, 40m) significantly discriminated starters from the non-starters, with the former recording superior scores. Except for the anthropometric findings, results reported for physiological characteristics in Gabbett^[7]^ were similar to results from other studies sharing the same definition of “starters”.^[10]^ In a different study involving 34 adults (22.7±3.4 years) playing Australian Rules Football, Young et al. ^[11]^ reported that starters had significantly greater 10 m speed and Yo-Yo Intermittent Recovery Level 2 (Yo-Yo IR L2) test scores compared to non-starters. For the rugby-specific game skills, Gabbett et al.^[12]^ showed that professional rugby league players selected to play in the first game of the season had superior tackling proficiency and dual-task draw and pass ability scores than non-selected players. Collectively, these results illustrate the possible influence of playing experience and performance measures on playing status in contact team sports of rugby league and Australian Rules Football, which are similar to rugby union.

The present study aimed to investigate differences in playing experience, anthropometric qualities, and performance measures (physiological characteristics and rugby-specific game skills) between elite U16 schoolboy rugby union starters and non-starters in the African context. The hypothesis was that starters would have superior scores for all the measures compared to the non-starters, except for anthropometric variables. These findings will facilitate an understanding of the qualities/skills considered by coaches for team selection and in need of further training in starters or non-starters.

## Methods

### Study design, setting and participants

This cross-sectional study examined the playing experience, anthropometric qualities, and performance measures of elite U16 schoolboy rugby players. These qualities or skills were chosen based on evidence from previous studies that they are likely amenable to training intervention.^[6–8,13]^ Seventy-one schoolboy rugby players were recruited from three randomly selected schools based in Harare, Zimbabwe, playing in the Super Eight Schools Rugby League (SESRL). The SESRL comprises eight schools known for playing highly competitive rugby (elite).^[3]^ The SESRL runs yearly for 13 weeks, from the beginning of May to the beginning of August. Throughout the season, each school plays home and away matches with the other schools in the league. Competitive matches were played every Saturday. In all the selected schools, Monday to Friday represent coach-led training days from 14h00 to 17h00. However, the weekly training structure varies across the SESRL schools depending on the coach’s training philosophies. Sundays are used as recovering/resting days for the schoolboys in preparation for Monday classes and rugby training.

### Eligibility criteria

All rugby players (n=90) for the U16 first team were invited to participate. However, only schoolboy rugby players with parental consent and had assented to participate were eligible. All the eligible players were categorised as “starters” or “non-starters”. At the time of data collection, the selected schools had played between six to eight competitive games out of the possible 14 games played in one SESRL season. Therefore, the operational definition of “starters” included all players who were part of the starting line-up for the U16 first team in at least 75% (n≥4) of the total competitive games played in the season. The threshold of ≥75% was logically adopted from a similar study conducted by Cabarkapa et al.^[14]^ and ensured that the starters had played most of the competitive matches. In Zimbabwe, coaches are mandated to offer every schoolboy rugby player, regardless of playing or performance status, an opportunity to play for the school team for fairness and inclusivity. However, to ensure that the recruited starters had satisfactory competitive game time, all eligible players had to have started and played at least 30 minutes in each competitive match. Furthermore, in an attempt to standardise training exposure across the selected schools, starters had to indicate having attended at least 80% of the total training days before testing. This information had to be corroborated by the head coaches. Based on self-report from the Physical Activity Readiness Questionnaire (PAR-Q) and medical information obtained from parents/guardians using the Adolescent Medical Health Questionnaire (AMH-Q), all eligible players had to be physically fit and free of medical conditions exacerbated by the performance of physical activities at the time of data collection.

### Ethical considerations

The research was ethically approved by the Medical Research Council of Zimbabwe (MRCZ ref: A/2070). Permission to access the schools and participants was obtained sequentially from the Ministry of Primary and Secondary Education, school headmasters, sports directors and U16 head coaches. Subsequently, parents/caregivers were approached for written informed consent for their child to participate in the study. Information letters were sent to the parents/caregivers explaining the aim of the study, significance of the study, reasons for including schoolboy rugby players, primary outcome variables to be measured and data collection procedures. Moreover, the research team scheduled face-to-face meetings with the parents/caregivers to further explain study procedures and implications for their child involvement. The schoolboy rugby players whose parents/caregivers consented had to provide a separate written informed assent before enrolment for testing.

### Instrument

Participants initially completed a questionnaire eliciting demographic and rugby-related information. The validated questionnaire sought age information, player position (*e.g. forward, backline*), specific team role (*e.g. hooker*, *fly-half etc*.), playing status (*regular starter vs. regular non-starter*), playing experience (*number of years playing competitive high school rugby*), in-season training exposure (*number of training days the players attended since SESRL started*), number of competitive matches played full time from the start of the SESRL up to the time of data collection and the approximate amount of time played in each competitive match *(≤30 minutes vs ≥30 minutes)*. Furthermore, the participants had to indicate whether they had started each competitive match they played or were substitutes.

### Test battery

The following outcome variables were included in the test battery: anthropometry (*height, sitting height, body mass*), physiological characteristics (*speed, agility, upper-and lower-muscular strength, upper-and lower-muscular power, muscle flexibility, prolonged high-intensity intermittent running ability*) and rugby-specific game skills (*tackling proficiency, passing ability, passing accuracy and catching ability)*. The development and validation of the constituent test items in the test battery have been presented elsewhere.^[15]^ However, the supplementary file provides a detailed description of each test. Briefly, speed was tested using a stopwatch for the linear distances of 10m, 20m and 40m. The L-run agility test, specifically modified for schoolboy rugby players, assessed change of direction speed (agility) using a stopwatch. The Sit-and-Reach (SR) test evaluated hamstring muscle flexibility using the sit-and-reach box. The Vertical Jump (VJ) test and 2 kg Medicine Ball Chest Throw (2 kg MBCT) evaluated lower and upper extremity muscular power, respectively. The Push Up test evaluated upper-body muscular strength/endurance, whilst the Wall Sit Leg Strength (WSLS) assessed unilateral static leg strength. The participants’ prolonged high-intensity intermittent running abilities were assessed using the Yo-Yo Intermittent Recovery Level 1(Yo-Yo IR L1). For the rugby-specific game skills, the Tackling Proficiency test assessed participant tackling abilities based on a specific technical criteria checklist. The Running-and-Catching Ability test evaluated catching skills. The Passing-for-Accuracy over 7m and Passing Ability skills tests assessed passing accuracy over a distance of 7m and passing technical abilities, respectively (supplementary file).

### Statistical analysis

The statistical analysis was conducted using Statistical Package of Social Sciences (SPSS) (Released 2021. IBM SPSS Statistics for Windows, Version 28.0. Armonk, NY: IBM Corp). The Shapiro-Wilk test examined the violation of the normality assumption for continuous variables. For normally distributed variables with equality of variance, the Independent Samples t-test compared the mean differences between the starters and non-starters (p<0.05). Hedges’ *g* values were used to indicate the magnitude of the effect size (ES), with 0.2, 0.4, and 0.8 interpreted as small, medium, and large effect sizes, respectively.^[16]^ The Chi-square test of association (χ^2^) assessed the relationship between player position differences by playing status categories (p<0.05). All continuous variables that showed statistical significance were inputted into a binary logistic regression model to determine significant predictors for playing status (starters vs non-starters). The assumptions for logistic regression of linearity and absence of multicollinearity were tested before analysis. The Pearson correlation analysis was used for collinearity diagnostics with a coefficient of >0.7 indicating minimal threshold for multicollinearity between the numerical predictor variables.

## Results

Table 1 compares the starters and non-starters in terms of age, playing experience, player position, training exposure, anthropometric features, and performance measures. The starters had more playing experience than non-starters (p<0.001, *g*=1.3). Additionally, starters showed superior scores for isometric/static strength of the lower legs (p=0.01, *g*=0.6), prolonged high-intensity intermittent running ability (p=0.03, *g*=0.5), tackling proficiency (p=0.02, *g=*0.8*)*, passing ability skill (p=0.01, *g*=0.7), and passing-for-accuracy over 7m (p<0.001, *g*=1.0) compared to non-starters.

Table 2 shows Pearson correlation coefficients for the variables significantly discriminating starters from the non-starters. Collinearity diagnostics showed a high correlation coefficient (*r*=0.7) between tests for passing ability and passing for accuracy over 7m. Consequently, passing for accuracy over the 7 m test was removed from further analysis.

The logistic regression model was statistically significant based on the Omnibus test of model coefficient χ^2^=38.6, *p*<0.001 and the Hosmer Lemeshow test, χ^2^=5.3, p=0.7. The model explained 57% (Nagelkerke *R^2^*) of the variance in playing status and correctly classified 83% of starters. As shown in Table 3, playing experience showed the best predictive ability (p<0.01). Participants with greater playing experience were 6.5 times more likely to be starters.

## Discussion

To the authors’ knowledge, this is the first study to determine whether differences exist between starters and non-starters of elite U16 schoolboy rugby union teams in an African context regarding playing experience, anthropometric features, and performance characteristics. The current evidence is largely from cross-sectional studies conducted using rugby league and Australian Rules Football participants from developed countries.^[7,9–13]^ However, differences in sports codes, junior rugby development, talent identification initiatives, rugby coaching philosophies, training exposure, competitive standards, player characteristics, socioeconomic status, and environmental and lifestyle-related factors affect the extrapolation of results between countries. All these factors have a combined interactive effect on schoolboy rugby players’ physiological characteristics and rugby-specific game skills. Therefore, evidence from a particular context may have limited practical application in a different setting, justifying the need for this study among Zimbabwean elite U16 schoolboy rugby union players.

The present study showed that elite U16 schoolboy rugby players selected as starters in most of the SESRL matches in Zimbabwe had greater playing experience than the non-starters. Participants with greater playing experience were 6.5 times more likely to be starters. Consistent with the present study findings, previous studies also highlighted the discriminative ability of playing experience in determining playing status.^[7,9,10,17]^ Although Gabbett^[7]^ had a different operational definition of “starters” to our study, the authors reported that starters of the first game of the competition had greater playing experience compared to the non-selected players among 53 adolescent rugby league players. Therefore, our study findings support the importance of schoolboy rugby playing experience in influencing playing status. The strong predictive ability of playing experience on playing status among Zimbabwean elite U16 schoolboy rugby players is unclear from the present study. However, since the participants had 2.4±0.6 years of coach-led playing experience, it is possible that U16 head coaches from the selected schools preferentially selected players they are historically aware of in terms of longitudinal/temporal performance from the previous age grades (U13, U14, and U15).

Another possible explanation is that, since playing experience represents a key factor in the development of qualities/skills needed in rugby largely because of increased training and match exposure^[18,19]^, it is possible that U16 rugby coaches from the selected schools strongly associate playing experience with competitive match success. Although the present study did not investigate that association directly, the significant correlation between playing experience and performance measures for lower-limb strength, endurance, tackling and passing (Table 2) provides a positive hint. These physiological qualities and rugby-specific game skills have been reported as essential for team success in rugby codes.^[1,3,5,6]^ However, these results call for further research to substantiate the reasons for starters having greater experience. Also, qualitative studies are needed in future to explore the perceptions of coaches for elite schoolboy rugby players on the factors that influence team selection and match success.

Out of the 12 physiological characteristics tested in the present study, only two (2) differentiated Zimbabwean elite U16 schoolboy rugby starters from the non-starters (Table 2). These results are both surprising and interesting at the same time. Surprising in the sense that, for the majority of the physiological variables tested (speed, flexibility, agility, and upper-body muscular strength/endurance) the group differences between starters and non-starters were non-significant. Contrary to our hypothesis, this indicates possession of similar cross-sectional qualities between the starters and non-starters in the Zimbabwean cohort of elite U16 schoolboy rugby players despite differences in playing experience. Previous studies such as the one conducted by Gabbett^[7],^ however, reported similar findings to the present study findings among U14 rugby league players for variables such as speed (10m, 20m, 40m), velocity (0–10 m/s, 10–20 m/s, 20–40 m/s), agility (change of direction speed test) and lower body muscular power (VJ test). The same study also showed that U18 rugby players selected as starters had scores similar to those of non-starters for speed, velocity, lower body muscular power, and maximal aerobic power. For the present study, these results may be explained by the fact that the sample had players playing elite schoolboy rugby matches in the SESRL; a competitive league which requires all players, regardless of playing status, to have similar levels of speed, agility, upper body muscular strength/endurance and flexibility. Reportedly, these qualities are relatively important in rugby^[1,5]^ and should be optimal for both starters and non-starters for safety and effective participation especially if both groups have similar training exposure days, chronological and biological maturation age, and anthropometry features (Table 1).

Interestingly, the main finding of this present study is that starters showed better scores for lower-limb muscular strength and endurance than the non-starters. The findings compare^[7,17]^ and contrast^[7]^ with other previously reported findings highlighting results variability and the controversial influence of physiological characteristics in determining playing status in rugby codes. For example, Gabbett^[7]^ found that scores for maximal aerobic power in terms of total distance (m) covered and predicted VO_2max_ (ml/kg/min) measured using the Multistage Fitness (MF) test were similar between starters and non-starters for the U16 and U18 age categories. This was in contrast to the present study findings. However, both scores were statistically different for the U14 players, with starters showing superiority. This latter finding is consistent with the present study findings, although differences in sport codes (*rugby union vs. rugby league*), age category (*U16 vs U14*) and test used (*Yo-Yo IR L1 vs MF*) could be highlighted between the two studies.

Although the present study failed to show a significant difference in training exposure (in-season training days) between starters (29.2±1.1 days) and non-starters (29.2±1.2 days), it is possible that differences in the training structure (periodisation), training intensity, volume, and type of training among selected schools and between starters and non-starters could account for the observed results. However, this study did not capture such variables. Therefore, it remains unknown whether the starters had any specialised training for endurance and lower-limb muscular power, unlike the non-starters. Therefore, future studies should further interrogate the differences in training intensity, volume and type of training between starters and non-starters playing for the U16 elite cohort group.

The following game skills separated the starters from non-starters with moderate to large effect size: tackling proficiency, passing ability and passing-for-accuracy over 7m. Starters showed better game technical skills compared to the non-starters. These findings add support to the generally accepted notion that game skills are important in rugby and discriminative of players^[12]^ Comparisons between studies are rendered difficult because of differences in the sample population and tests used to evaluate game skills. Moreover, there are limited studies specifically comparing starters and non-starters of elite schoolboy rugby with regard to game skills in literature. Although Gabbett et al^[12]^ had professional rugby players in the sampling frame, the authors consistently reported that players selected to play in the season’s first game had superior tackling proficiency and dual-task draw and pass ability than non-selected players. However, for the present study, further investigations are still warranted to account for the differences between the starters and non-starters with regard to tackling and passing ability. Partaking in regular competitive matches possibly provided an extra advantage to starters for tackling and passing.

### Limitations

In spite of study novelty, the study findings should be interpreted cautiously. The findings represent cross-sectional differences between starters and non-starters derived from only one (1) league (SESRL) and one (1) age category (U16). This design, together with the relatively small sample size utilised (n=71), limits the external validity of the findings to other age groups and to the rest of the schoolboy players playing U16 rugby in sub-elite or amateur leagues. These findings are sufficient to serve as preliminary results for larger repeat studies in the future, aimed at confirming the relative importance of playing experience, anthropometry, and performance measures on playing status using robust study designs. Additionally, data collection was conducted during the competitive season when only six to eight games had been played. This limited the operational definition of starters to players who started at least 75% of the games played. Although this definition was pragmatic and contextually relevant, it failed to consider possible changes in players’ performance or playing status across the entire season.

This study only investigated anthropometric features and performance measures as potential factors influencing playing status. Although these factors are significant and amenable to training intervention, there could be a complex interaction of multiple factors such as tactical, attitudinal, behavioural or psychological influencing selection into the starting line-up of an elite U16 schoolboy rugby team. This is an important factor for consideration in future research studies comprehensively exploring differences between starters and non-starters in elite schoolboy rugby union players. Additionally, due to the cross-sectional nature of the study and limited data collection time, the utilised research approach fails to account for the potential influence of training-related variables such as type of training, structure, intensity or frequency on the performance measures across the three selected schools. This area requires further research and exploration in schoolboy rugby players.

## Conclusion

This study showed that starters had greater playing experience than non-starters. Practically, these findings inform coaches and scouts to consider playing experience when selecting schoolboy rugby players to represent Zimbabwe in the U16 national team against international teams. Additionally, the starters outperformed the non-starters in lower-limb isometric strength, prolonged high-intensity intermittent running ability, tackling proficiency, passing-for-accuracy over 7 m and passing ability skill. This finding suggests that coaches and scouts should consider important differentiating qualities/skills during talent identification initiatives, player recruitment, and team selection for U16 teams. From a practical perspective, schoolboy rugby coaches should incorporate specific training drills or routines involving repeated high-intensity performance exercises, repeated sprinting abilities or prolonged high-intensity intermittent running ability aimed at facilitating the development and maintenance of endurance, lower-leg muscular strength, tackling proficiency and passing ability skill, especially among U16 schoolboy rugby players considered as non-starters. This will ensure they have optimal physiological attributes and exceptional rugby-specific game skills like the regular starters. Specifically, coaches can deliberately increase the training intensity or frequency for lower-limb muscular strength, endurance, tackling and passing for the non-starters and offer them commensurate game time for a competitive stimulus. However, for most physiological characteristics such as speed, agility, flexibility, and upper-body muscular strength/endurance, there were no differences between starters and non-starters. Cognisant of the study limitations, these findings further highlight to the coaches the important qualities/skills all strength and conditioning coaches should focus on for longitudinal training and player development among U16 schoolboy rugby union players regardless of playing status.

## Supplementary Information



## Figures and Tables

**Table 1:** Differences between starters and non-starters (n=71)

Variables	All players (n=71) Mean±SD	Starters (n=35) Mean±SD	Non-starters (n=36) Mean±SD	Test statistic	p-value

Chronological age (years)	14.9±0.4	14.9±0.4	14.8±0.4	t (69)=0.9	0.4
Predicted APHV (years)^*a*^	14.7±1.0	14.6±0.9	14.8±1.1	t (69)=−0.9	0.4
Playing experience (years)	2.4±0.6	2.7±0.5	2.06±0.5	t (69)=5.9	**<0.001** ^++^

**Anthropometry**					

Body mass (kg)	62.6±12.2	64.0±15.2	61.2±8.2	t (69)=0.9	0.3
Height (m)	1.7±0.1	1.68±0.1	1.67±0.1	t (69)=0.9	0.4
BMI (kg/m^2^)	22.5±2.9	22.8±3.5	22.2±2.3	t (69)=0.8	0.4
Sum of seven skinfolds (mm)	67.1±25.3	68.4±31.9	65.8±16.9	t (69)=0.4	0.7

**Playing positions**					

Forward, n(%)	36(51)	17(49)	19(53)	X^2^=0.1	0.7
Backline, n(%)	35(49)	18(51)	17(47)		

**Training exposure**					

In-season training days	29.2±1.2	29.2±1.1	29.1±1.2	t (69)=0.3	0.8

**Competative matches played**					

Number of games (n)	3.4±1.9	5.2±0.6	1.7±0.6	t (69)=−24.4	**<0.001**

**Physiological tests**					

10m speed (s)*	2.2±0.2	2.3±0.2	2.2±0.1	t (65)=0.4	0.7
20m speed (s)*	3.5±0.2	3.5±0.3	3.5±0.2	t (65)=−0.2	0.8
40m speed (s)*	6.2±0.5	6.2±0.6	6.2±0.4	t (65)=−0.3	0.8
Modified L-run (s)*	6.5±0.4	6.5±0.5	6.6±0.3	t (65)=−0.4	0.7
Sit-and-Reach (cm)***	5.7±4.9	5.9±5.5	5.5±4.3	t (68)=0.4	0.7
Vertical Jump (cm)	36.9±3.1	36.9±3.4	36.8±2.8	t (69)=−0.1	0.9
2kg MCBT (m)	6.5±1.0	6.4±1.0	6.7±0.8	t (69)=1.4	0.2
20s Push Up (n)**	20.6±3.3	21.0±3.6	20.3±3.0	t (67)=−0.9	0.4
60s Push Up (n)**	37.3±10	38.1±11.3	36.5±7.9	t (69)=−0.7	0.5
Time to Fatigue (s)**	42.0±9	41.9±10.3	42.0±7.6	t (69)=0.0	1.0
Wall Sit Leg Strength (s)	128±16	133±13	123±18	t (69)=2.7	**0.01** ^+^
Yo-Yo IR L1 (m)	1 190±281	1 264±297	1 119±248	t (69)=2.2	**0.03** ^+^

**Technical skills tests** ***					

Tackling Proficiency (%)	78±11	80±12	74±9	t (68)=2.4	**0.02** ^+^
Passing Ability Skill (au)	105±5	107±5	104±4	t (69)=2.8	**0.01** ^+^
Passing-for-Accuracy (%)	75±8	79±8	72±6	t (69)=4.1	**<0.001** ^++^
Running-and-catching ability ^]^ (au)	70±3	71±3	70±3	t (69)=1.2	0.2
Tackling Proficiency (%)	78±11	80±12	74±9	t (68)=2.4	**0.02** ^+^

Bold text indicates significant values of p<0.05.

* (n=67);

** (n=69);

*** (n=70);

BMI, body mass index; mm, millimetres; s, seconds; 2kg MBCT-2kg medicine ball chest throw; Yo-Yo IR L1-YoYo intermittent recovery level 1; au-arbitrary scores; M±SD, mean±standard deviation; %, passing for accuracy, The Passing-for-Accuracy over 7 m test; playing experience represents the number of years the participants have been playing schoolboy rugby since admission at the school; training exposure-number of training the participants partook in training. Hedges’ g effect size values were large (++) for passing for accuracy over 7 m test and playing experience and moderate (+) for wall sit leg strength, Yo-yo IRL1, Tackling, and passing ability skill tests (a); APHV, Age at Peak Height Velocity^*a*^ was deduced from this equation: −9.326 + (0.0002708 x [leg length x sitting height]) − (0.001663 x [age x leg length]) + (0.007216 x [age x sitting height]) + 0.02292 x [weight/height].

**Table 2:** Pearson correlation coefficients for variables entered into logistic regression model

	Playing experience (years)	Wall sit leg strength test (s)	Yo-yo irl1 test (m)	Tackling proficiency (%)	Passing ability skill test (au)	Passing-for-accuracy test (%)
Playing experience	1.0	0.4*	0.3*	0.4*	0.2	0.4*
Wall Sit Leg Strength test		1.0	0.0	0.0	0.0	−0.0
Yo-Yo IRL1 test			1.0	0.5*	0.3*	0.4*
Tackling proficiency test				1.0	0.3*	0.4*
Passing Ability Skill test					1.0	**0.7** *
Passing-for-Accuracy test						1.0

* correlation co-efficients significant at 0.01 level (2 tailed);

Yo-Yo IR L1, YoYo intermittent recovery level 1; au, arbitrary scores

**Table 3:** Binary logistic regression results for variables predictive of playing status

Independent variables	B	SE	Wald	Df	p-value	Exp (B)	95% CI Exp(B)	
							Lower	Upper
Rugby experience (years)	1.9	0.7	8.3	1	<0.01	6.5	1.8	23.3
Yo-Yo IRTL (m)	0.0	0.0	2.4	1	0.1	1.0	1.0	1.0
Tackling proficiency test (%)	0.0	0.0	0.1	1	0.8	1.0	0.9	1.1
Passing Ability Skill test (au)	0.2	0.1	5.0	1	0.03	1.2	1.0	1.5
Wall Sit Leg Strength test (s)	0.1	0.0	6.8	1	0.01	0.9	0.9	1.0
Constant	19.9	8.9	5.0	1	0.03	0.0		

Yo-Yo IRTL (m), Yo-yo intermittent recovery level 1 test; 95% CI, confidence interval; df, degrees of freedom; SE, standard error of regression
