# Supplementary material for: Differences in playing experience, anthropometry and performance measures between Under 16 schoolboy rugby players classified as starters or non-starters: A comparative cross-sectional study
Source: S Afr J Sports Med. 2025 Jun 15;37(1):v37i1a19947. doi: 10.17159/2078-516X/2025/v37i1a19947 (PMC12200344; doi:10.17159/2078-516X/2025/v37i1a19947)
Supplement: Supplementary file 1 [file 2078-516X-37-v37i1a19947-s001.pdf]

Differences in playing experience, anthropometry and performance measures between Under 16 schoolboy rugby players classified as starters or non-starters: A comparative cross-sectional study

Test Battery

Height: Standing height was measured using a portable stadiometer. The testing procedure involved lowering the stadiometer to the top of the participant's head and recording the height to the nearest 0.1cm. The mean of the two measurements was recorded. The intraclass correlation coefficient (ICC) for height was 0.9 with a coefficient of variation (CV) of 0.6%.

Body mass: The body mass was measured, to the nearest 0.1kg, using a calibrated digital scale. Instructions were to stand still and arms hanging by the body sides. The mean of the two measurements was recorded. The ICC for body mass was 0.9 with the CV of 0.7%. Body mass and height scores were used to calculate the body mass index (BMI) of each participant using Quetelet index (kg/m^2).

Skin fold measures: The sum of seven site skinfolds *(Biceps, Triceps, Subscapular, Suprailiac, Abdominal, Thigh and Calf)* was calculated as a measure of body composition using the Lange skinfold callipers. Instructions were to stand in anatomical position with shirt removed. Participants were measured on the right side of the body regardless of dominance. The general procedure involved "pinching" the skin and the subcutaneous fat between the thumb, ring and middle finger. The skinfold calliper would be placed 1cm below the finger, deep into the skinfold. Two measurements per site were taken and had to agree within 1 millimetre (mm). The mean of the two measurements was recorded for each site. The ICC for sum of skin fold measures was 0.9 with a CV of 4.3%.

Linear speed: The linear speed was measured based on the 10m, 20m and 40m speed test. Participants wore light clothes and run in their rugby boots. Participants ran the 10m, 20m and 40m distance in that order. Time taken was measured by a digital stopwatch. Two test trials were run for each distance and the better score was recorded. The ICC (CV %) for the10m sprint time was 0.9 (2.10), for 20m sprint test was 0.9 (1.9) and for 40m sprint test was 0.9 (1.2).

Agility: A modified L-run agility test assessed the agility. Three cones were placed 5 m apart to represent an 'L' shape. Participants started in prone position, mimicking a rugby player coming from a physical contestation. Instructions were to get up, pick a rugby ball at the first cone and run fast along the 5 m, turn left, run forward in between the cones, turn 180 degrees, and then run straight to the finish line. The test was completed when the rugby ball was dropped at the starting line. Time run was recorded using a stopwatch. Two trials were run and best count recorded in seconds. The ICC (CV%) for the test was 0.9 (2.5).

Lower-leg muscular power: The VJ test measured isotonic lower-leg muscular power. Participants wore light clothes and no shoes. Instructions were to stand feet flat with the wall lateral to player's dominant side. Firstly, participants extended their upper extremity and mark the highest possible point on the wall with the chalk (standing height). Secondly, players crouched to 90 degrees of knee flexion holding the chalk between the index and middle finger. Afterwards, participants had to spring upward as high as possible and mark the highest point possible on the wall (jump height). The difference between the jump height and standing height was measured using a tape measure. Two measurements were taken with the best count recorded in centimetres. The ICC (CV%) =0.9 (2.2).

Lower-leg muscular strength: The modified Wall Sit Leg Strength (WSLS) evaluated the isometric strength of the dominant lower extremity. Instructions were to stand comfortably, feet shoulder width apart, back and head against a vertical wall. Participants slided down maintaining contact with the wall until both knees and hip are at 90°. However, time started when the non-dominant leg was lifted off ground and kept extended. The test stopped when participants failed to maintain the position or the extended leg returned to the ground. Two measurements were taken with the best count recorded in seconds. The ICC (CV%) for the test was 0.9 (3.1).

Upper-limb muscular strength, endurance and time-to-fatigue: The 20 seconds (20s) Push Up and 60 seconds (60s) Push Up tests assessed upper-body muscular strength and endurance, respectively. Participants began in prone, with hands on the floor, thumbs shoulder-width apart and elbows fully extended. Instructions were for participants to descend to the researcher fist placed on the ground and then ascend until the elbows were straight. Participants performed the maximum number of push-ups quickly in 60s. The number of push-ups performed in 20s measured upper-body muscular strength whilst the number done in 60s measured upper-body muscular endurance. The ICC (CV%) for the 60s Push Up test was 0.94 (2.87). The drop-out time was also recorded as Time-to-Fatigue for participants who failed to complete the full 60 seconds.

Upper-body muscular power: The 2kg Medicine Ball Chest Throw (2kg MBCT) assessed upper-body muscular power. Participants threw a 2kg medicine ball as far as possible while seated with the back, and legs straight. Distance was measured using the tape measure. Two trials were performed recording the better score. The ICC (CV%) for the test was 0.91(1.45).

Prolonged high-intensity intermittent running ability: The Yo-Yo Intermittent Recovery Level 1 (Yo-Yo IRL1) test evaluated prolonged high-intensity intermittent running ability of players. A pre-test warm procedure and practice trails were conducted first. Instructions were to run back and forth along a 20m track, keeping in time with a series of signals on a compact disk. The frequency of the audible signals (and hence running speed) was progressively increased until subjects reach volitional exhaustion. The total distances covered in meters was recorded. The ICC (CV%) for the test was 0.92 (2.94).

Muscle flexibility: The Sit-and-Reach (SR) test evaluated the flexibility of the lower back and hamstring muscles. Participants sat on the ground, knees extended, feet contacting the rigid SR box. Instructions were to reach forward as possible with stacked hands and to hold that position for one second. Two attempts were made and the best score recorded. The ICC (CV%) for the test was 0.88 (4.67).

Tackling proficiency test: A simulated rugby-specific match 2-on-1 scenario was simulated within a 10 × 10 m grid for the tackling test (Figure 1). A local rugby coach with experience served as an expert rater, Observer 1 (Obs 1) for the skill rating of the participants. The players performed a warm-up led by the lead author, which consisted of jogging and upper and lower body dynamic stretching. Three players were used at one given time. The two attacking players (ball carriers) were former Under 19 rugby players recruited as research assistants for this test. They were instructed to advance from one side of the grid to the other and complete one pass each before being tackled by the defending player (test player). The test players were oblivious of the number of passes to be completed between the attacking players to mimic a real rugby scenario which is underlined by unpredictability. The attacking players were set at 4m apart from each other and 5m away from the tackler at the beginning. The attacking players had to make passes between them within the 3m while advancing forward. A line at 3m indicated where no more passes between the two attacking players were allowed. The test player was initially stationed 5m away from the straight line, marking the starting line of the attacking players. The procedure was for the test player to tackle the player with the ball. If the attacking player runs well that the test player does not get him, the test was repeated. Demonstrations were given to enhance players understanding of the test and to provide them with a reference for the required match-like intensity. After one cycle of this protocol, the players waited for a brief recovery period (1 minute) on their feet at the opposite end of the grid before repeating the drill. Six test trials were conducted to allow the observer to observe the tackling skills under fatigue as well.

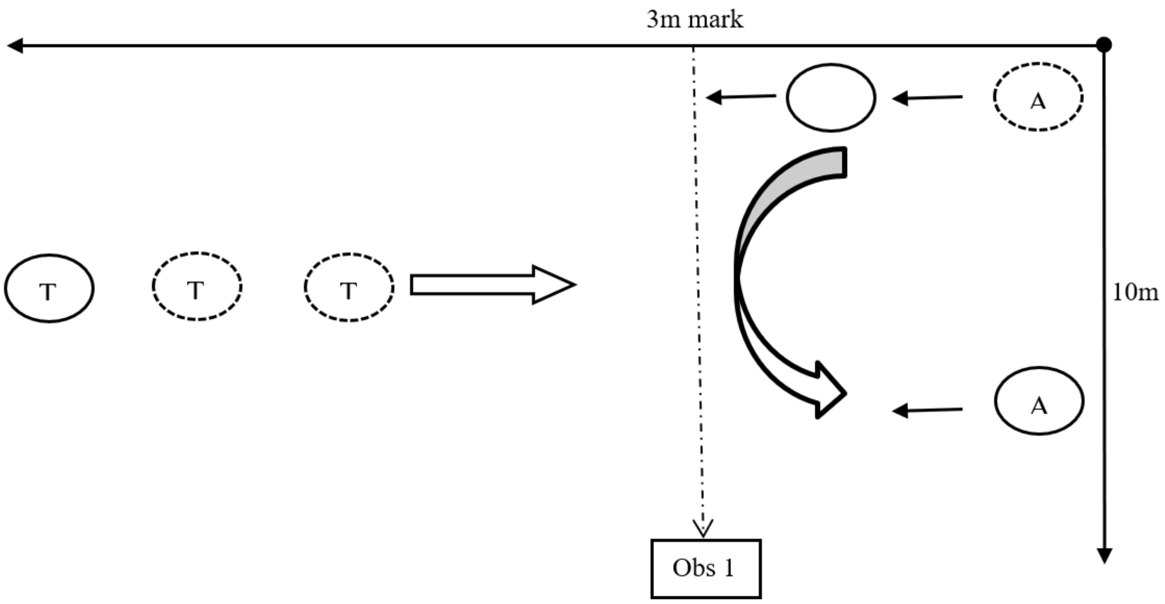

Key

T- Tackling Player Being Assessed; A-Passing and Receiving Player; Obs 1-Observer 1 =Ball passing direction

Figure 1. *Illustration of the tackling protocol*

The observer assessed and scored each of those six trials based on technical checklist. The observer rated each player in real time on their overall proficiency in each skill using a Likert scale (*0-not achieved completely; 1-partly achieved; 2-completely achieved relevant criteria)*. The coaches were given the assessment criteria two weeks prior to testing, and the details were discussed with them. The technical criteria were as follows:

1. *Contacting the target in the centre of gravity/low body position*
2. *Contacting the target with at least one shoulder*
3. *Head to one side of the body*
4. *Arms should readiness for tackling*
5. *Body position square/aligned*
6. *Arms completely wrapped around the target on contact when tackling*
7. *Leg drive on contact/ drive with the legs*
8. *Centre of gravity forward on base of support*
9. *Maintain grip until the attacking player is on ground*
10. *Turtle the player/ hold the player immobile on the ground/defensive shape*

***Passing-for-accuracy over 7m test and passing ability skill test*:** This test was used for combined assessment of passing skill ability and accuracy in passing for over a 7m distance. Only one player was tested at any given time. Participants commenced chest down,

flat on the ground, and knees extended behind the starting line (Figure 2). They stood up on the word "go" from a research assistant stationed at the starting line and grabbed a rugby ball placed on the touchline, and sprinted in a zig-zag way on a 10m course set out using cones. The participants were instructed to run as quickly as possible. Upon entering the passing grid zone (measuring 3m x 3m) they were supposed to release the rugby ball and prepare to receive a pass from one of the expert coaches (E1) acting as a research assistant for the study. They were instructed to catch the ball and pass immediately at a moving target (R) placed at a 7m distance with a defensive player approaching to offer a hindrance (A). The pass had to be made from the centre of the passing grid zone. Another rugby expert (E2) rated the pass made by the first expert (E1) and if the pass was deemed bad, the test was repeated. The target (R) would have started at the starting line and moved slowly with the pace of the tested player in anticipation of receiving the ball. The target player used was former U19 elite rugby player acting as a research assistant for the research team. After each pass, the subject ran back to the starting line and repeats the test without starting in prone this time. They started each 10 m sprint at a 20 sec cycle. This happened five times, making it one set of passing ability assessments. The second set started 60 seconds after the completion of the first set with the participant in prone again, and chest on the ground and repeating everything else alluded above. After completing the second set of five runs, the participant rested again for 60 seconds before embarking on the last final set. Overall, the total passes made were 15 executed in three sets of 5. Participants decided which side they preferred to pass depending on their hand dominance. The number of accurate passes made (passes caught) to the receiver (R) was determined by the lead author observing and expressed as percentage of the total passes made to give the passing accuracy (%) score for the participant. In addition, an expert rugby coach (E2) judged passing ability looking at the eight elements giving a passing ability score for each pass made looking at the technical elements utilised in the pass. The scoring was based on a dichotomous response scale: *0-not achieved; 1-achieved*. So, each participant was assessed 15 times (3 sets of 5) and a score was recorded for each technical element. All the scores were then added for each test trial to give a total passing skill score reflecting passing ability score in arbitrary units. The technical criteria assessed:

1. *Pendulum action*
2. *Looking where pass is to be made*
3. *Single movement*
4. *Straight follow through of passing hand*
5. *Appropriate ball speed*
6. *Pass in front of the receiver*
7. *Receiver catches the ball*
8. *Receiver maintain stride/minimal breaking of the receiver pace to receive the ball*

***Running-and-catching ability test**:* The protocol was more or less similar to the passing protocol and was mainly designed for the assessment of catching ability skills test based on expert rating. Briefly, participants ran in a zigzag fashion for 10m holding the rugby ball (Figure 3). Starting position of prone was similar as for the passing procedure. Upon entering the catching grid zone (measured 3m by 3m) the ball is passed immediately from 7 m and the test player should show ability to catch the ball. The test was performed on a different day from the passing protocol to allow for independent assessment of the skills accurately. Two player assistants providing defensive play were utilised in the protocol. Their duty was to provide cognitive recognition of impending attack to the test player for the passed ball to simulate match rugby situations. Their hidden aim was to compete for the ball as well with the tested

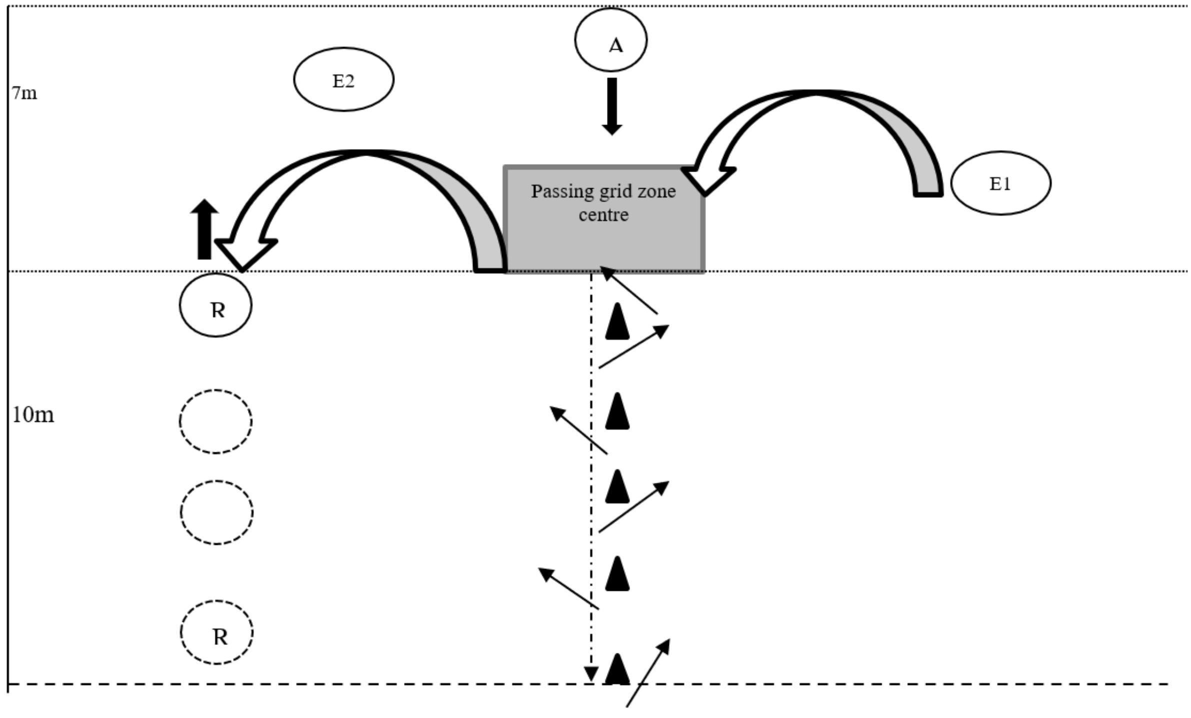

R, Receiver; A, Attacking player; E1, Coach Expert 1(Passer); E2, Coach Expert 2 (Rater), ▲, cone

Figure 2. *Passing protocol for passing ability and accuracy assessment*

player. The two assistants were placed equidistant from participant catching grid. There were also two coach experts acting as research assistants, one rated the technical ability of catching as a skill and the other one was helping with the decision on the quality of the pass made to the test participant. If the pass was deemed not suitable, looking at some of the key things highlighted under passing ability, then that pass will not be rated for catching. Otherwise, a re-throw was done. If the pass was deemed acceptable and the test participant misses it was recorded as a missed catch and awarded corresponding scores. There were technical criteria used for assessment of running-and-catching ability. Each of the five criteria was assessed based on a Likert scale from 0 (failed completely to perform the activity), 1 (completely achieved). Participants were assessed 15 times (3 sets of 5) just like in the passing protocol. The idea is to see how the participants would fare before and after fatigue has set in. The total score per test trial was 5 aggregating to 75 after completion of 15 tests trial. The technical criteria looked at the following elements:

1. *Eyes on the ball/Focus on passer/ Body receptive to the pass*
2. *Hands up/elbows bent/*
3. *Fingers spread/palms out and thumbs up*
4. *Take the pass early/meet the ball early*
5. *Catch the pass/Hold the "body" of the ball and all this with minimal breaking of the natural or starting stride of the player.*

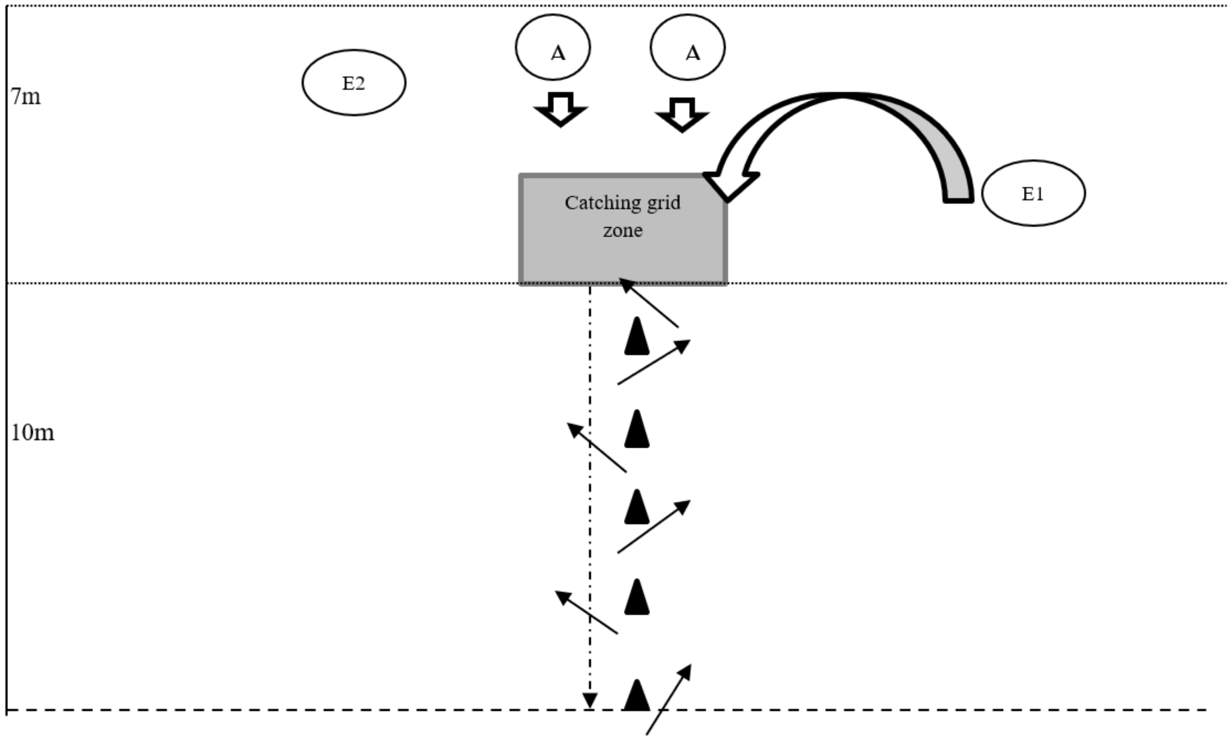

Key: A, Attacking players; E1, Coach Expert 1(Passer); E2, Coach Expert 2 (Rater)

Figure 3. *Running-and-catching protocol for assessment of catching ability skills*
